# Highly Thermostable Xylanase Production from A Thermophilic *Geobacillus* sp. Strain WSUCF1 Utilizing Lignocellulosic Biomass

**DOI:** 10.3389/fbioe.2015.00084

**Published:** 2015-06-16

**Authors:** Aditya Bhalla, Kenneth M. Bischoff, Rajesh Kumar Sani

**Affiliations:** ^1^Department of Chemical and Biological Engineering, South Dakota School of Mines and Technology, Rapid City, SD, USA; ^2^Renewable Product Technology Research Unit, Agricultural Research Service, National Center for Agricultural Utilization Research, U.S. Department of Agriculture, Peoria, IL, USA

**Keywords:** biofuels, corn stover, xylanase, prairie cord grass, thermostable, untreated lignocellulose

## Abstract

Efficient enzymatic hydrolysis of lignocellulose to fermentable sugars requires a complete repertoire of biomass deconstruction enzymes. Hemicellulases play an important role in hydrolyzing hemicellulose component of lignocellulose to xylooligosaccharides and xylose. Thermostable xylanases have been a focus of attention as industrially important enzymes due to their long shelf life at high temperatures. *Geobacillus* sp. strain WSUCF1 produced thermostable xylanase activity (crude xylanase cocktail) when grown on xylan or various inexpensive untreated and pretreated lignocellulosic biomasses such as prairie cord grass and corn stover. The optimum pH and temperature for the crude xylanase cocktail were 6.5 and 70°C, respectively. The WSUCF1 crude xylanase was found to be highly thermostable with half-lives of 18 and 12 days at 60 and 70°C, respectively. At 70°C, rates of xylan hydrolysis were also found to be better with the WSUCF1 secretome than those with commercial enzymes, i.e., for WSUCF1 crude xylanase, Cellic-HTec2, and AccelleraseXY, the percent xylan conversions were 68.9, 49.4, and 28.92, respectively. To the best of our knowledge, WSUCF1 crude xylanase cocktail is among the most thermostable xylanases produced by thermophilic *Geobacillus* spp. and other thermophilic microbes (optimum growth temperature ≤70°C). High thermostability, activity over wide range of temperatures, and better xylan hydrolysis than commercial enzymes make WSUCF1 crude xylanase suitable for thermophilic lignocellulose bioconversion processes.

## Introduction

Lignocellulosic agricultural and forestry waste materials are the key substrates for second generation biofuels. Lignocellulose contains 20–40% of hemicellulose, which is a branched heteropolymer consisting of pentose (D-xylose and D-arabinose) and hexose (D-mannose, D-glucose, and D-galactose) sugars with xylose being most abundant (Cano and Palet, 2007; Kumar et al., 2008). Hemicelluloses are classified according to the main sugar in the backbone of the polymer, e.g., xylan (β-1,4-linked xylose) or mannan (β-1,4-linked mannose) (Jørgensen et al., 2007). To obtain these linked sugars, there is a need to effectively break the locked polysaccharides from recalcitrant lignocellulose. There have been various reports published on deconstruction of hemicellulose utilizing hemicellulases (Gao et al., 2011). Among hemicellulases, endoxylanases and β-xylosidases are reported to be the main components responsible for effective conversion of xylan fraction of biomass to monomeric xylose (Qing and Wyman, 2011; Bhalla et al., 2014a,b).

Thermophiles have often been proposed as a potential source of industrially relevant thermostable enzymes (Turner et al., 2007; Viikari et al., 2007). For example, thermophilic microbes of various genera, including *Bacillus*, *Geobacillus*, *Acidothermus*, *Cellulomonas*, *Paenibacillus*, *Thermoanaerobacterium*, *Actino-madura*, *Alicyclobacillus*, *Anoxybacillus*, *Nesterenkonia*, and *Enterobacter* have been reported to produce thermostable xylanases (Bhalla et al., 2013). Thermostable enzymes have an obvious advantage as catalysts in the lignocellulose conversion processes due to better enzyme accessibility and cell-wall disorganization achieved at high-temperature reaction conditions (Paes and O’Donohue, 2006). Also, high temperature allows better solubility of reactants and products by lowering the viscosities, leading to faster hydrolysis (Viikari et al., 2007). Longer active life under high temperature conditions would make these enzymes favorable for enhanced and efficient biomass conversion. Therefore, to be an effective enzyme, thermostability is the most important attribute for the enzyme utilized under extreme bioprocessing conditions. The present work describes the characterization of highly thermostable endoxylanases (simply referred to as crude xylanase cocktail) produced by *Geobacillus* sp. strain WSUCF1. Hydrolytic activity of WSUCF1 crude xylanase cocktail was studied, and compared to commercially available enzyme cocktails.

## Materials and Methods

### Microorganism, medium, and inoculums

The WSUCF1 strain was identified as *Geobacillus* sp. and affiliated to phylum *Firmicutes* by 16S rDNA analysis (Rastogi et al., 2010). The WSUCF1 strain was grown at 60°C and pH 7.0 in a minimal medium supplemented with xylan (0.2%) or lignocellulosic substrates (1%) as carbon and energy source. The composition of the medium per liter: 0.1 g nitrilotriacetic acid, 0.05 g CaCl_2_·2H_2_O, 0.1 g MgSO_4_·7H_2_O, 0.01 g NaCl, 0.01 g KCl, 0.3 g NH_4_Cl, 0.005 g methionine, 0.2 g yeast extract, 0.01 g casamino acid, 1.8 g of 85% H_3_PO_4_, 1 ml FeCl_3_ solution (0.03%), and 1 ml of Nitsch’s trace solution (Rastogi et al., 2009). Five percent of pre-culture grown was used to inoculate 100 ml of minimal medium containing carbon source in 500 ml Erlenmeyer flasks. The flasks were incubated in a shaker incubator at 60°C, 150 rpm for 96 h. Control flasks contained only carbon source without WSUCF1 cells, for each experiment. After every 12 h, 1 ml samples were removed aseptically, and analyzed. Growth was checked by measuring absorbance at 600 nm. Immediately after collection, the samples were centrifuged at 4°C and 10,000 × *g* for 10 min. Supernatant was analyzed for the endoxylanase activity as described below.

### Enzyme assay

The reaction mixtures contained 0.5 ml of 1% (w/v) Birchwood xylan (Sigma-Aldrich) in phosphate buffer (100 mM, pH 6.5) and 0.5 ml of an appropriate dilution of enzyme. The enzyme–substrate reaction was carried out at 70°C for 10 min and reaction was stopped by the addition of 1.5 ml 3,5-dinitrosalicylic acid (DNSA) solution, boiled for 10 min, and then cooled on ice for color stabilization. The optical absorbance was measured at 540 nm and the amounts of liberated reducing sugars (xylose equivalents) were estimated against a xylose standard curve.

### Parametric optimization for xylanase cocktail production

Physical parameters for crude xylanase cocktail activity production were optimized by maintaining all factors constant except the one being studied. Effect of pH on enzyme production was assessed by cultivating the WSUCF1 strain in non-buffered growth media of pH 5.0–9.0, at 60°C for 96 h. The pH of the medium was adjusted using 5M NaOH or 6N HCl. The effect of temperature was studied by growing the microbe at different temperatures (50–80°C), pH 7.0 for 96 h. Effect of various easily available inexpensive carbon and energy sources including untreated prairie cord grass (PCG) 1%, untreated corn stover (CS) 1%, thermo-mechanically pretreated prairie cord grass (PPCG) 1%, or thermo-mechanically pretreated corn stover (PCS) 1% was studied for crude xylanase activity. Growth conditions for the WSUCF1 utilizing these substrates are described above under Section “Microorganism, Medium, and Inoculums.” Thermo-mechanical pretreatment of the CS and PCG was carried out in a single-screw extruder as described earlier (Kannadhason et al., 2009).

### Characterization of the crude xylanases

Sodium dodecyl sulfate-polyacrylamide gel electrophoresis (SDS-PAGE) was performed as described by Laemmli (1970). Ten milliliters of supernatant from each growth flask containing different substrates (xylan, CS, PCS, PCS, and PPCG as substrate) were passed through Amicon Ultra-15 – Millipore (3 kDa cut-off) to 10× concentrate the protein. To obtain zymogram of crude endoxylanase and β-xylosidase activity following SDS-PAGE, samples (10 μl of concentrated protein) were mixed with 10 μl 2× SDS sample buffer, and heated to 95°C for 1 min prior to loading on gel. For endoxylanase, sample was loaded onto a 12% SDS-PAGE gel containing 0.1% (w/v) oat spelt xylan (OSX) polymerized within the gel matrix whereas for β-xylosidase, gel was separately incubated with the substrate for 30 min, i.e., 0.1 mg/ml 4-methylumbelliferyl-β-d-xylopyranoside in phosphate buffered saline, pH 5.9. Current (150 V constant voltage) was passed through the gel until the bromophenol blue dye-front migrated to the bottom of the gel. The gel was washed successively with the following for 30 min each: 20% isopropanol in phosphate buffer saline (PBS, 100 mM, pH 5.9), 8M urea in PBS, and PBS (pH 5.9) three times. For endoxylanase, the gel was incubated in PBS (pH 5.9) overnight at 37°C. The gel was stained with Congo Red (1 mg/ml) for 30 min, and de-stained with 1M NaCl in PBS until clear bands indicating xylanase activity were visible. For the detection of β-xylosidase activity, gel was examined for fluorescence on a UV light box indicating β-xylosidase activity.

The relative xylanase activity using 1% (w/v) Birchwood xylan was determined at various pHs. The pH optimum of crude xylanase was estimated by carrying out the reactions in the pH range of 3.0–10.0 using different assay buffers, citrate buffer (50 mM, pH 3–6), phosphate buffer (50 mM, pH 6–7.5), Tris-HCl (50 mM, pH 7.5–9), and glycine–NaOH buffer (50 mM, pH 8.6–10) at 70°C for 20 min. The enzyme activity obtained at the pH optimum was used to calculate the relative enzyme activity at other pHs. The optimum pH of 6.5 was used to determine the optimum temperature for the crude xylanase. The optimal temperature for crude xylanase was obtained by performing the enzyme assays at different temperatures. The experiments were carried out in the temperature range of 40–90°C under assay conditions as described above.

Thermostability of xylanases was assessed by incubating the enzyme at different temperatures 50–100°C with increments of 10°C for a period of 19 days. Subsamples were removed at definite time intervals over the period of incubation. The residual activities were determined under optimum pH and temperature conditions using the DNSA method as described above. In all cases, the initial activity was assumed to be 100% and used to calculate the enzyme activities as percentages of the initial activity during the incubation period.

### Hydrolysis of birchwood xylan

The hydrolysis of Birchwood xylan was carried out in 100 ml conical flask containing 50 ml sodium phosphate buffer (50 mM, pH 6.5), 1 g xylan, 0.03% (w/v) sodium azide, and 20 U xylanase/g xylan. The hydrolysis was performed for 48 h at different temperatures (50, 60, and 70°C) with a rotating speed of 150 rpm. Hydrolysis of xylan was also compared using commercial enzyme mix, Cellic HTec2 (Novozymes) and Accellerase XY (Genencor). Optimum pH of 5.0 was used for commercial enzymes to compare their hydrolytic potential at different temperatures. The amount of reducing sugar was measured by using DNS method as described above.

## Results and Discussions

Thermophilic bacteria are excellent source of thermostable xylanases, which have the potential to be utilized in lignocellulose hydrolysis (Bhalla et al., 2013, 2014a,b). Thermostable xylanases, optimally active at high temperatures and wide range of pHs are useful under harsh industrial processing conditions (Turner et al., 2007). The production of total extracellular proteins and enzymes when WSUCF1 was grown on xylan is shown in Figure S1A in Supplementary Material. In general, the increase in enzyme activity is associated with an increase in total extracellular protein. The WSUCF1 strain produced maximum crude xylanase activity (14.6 U/ml) on day 4 when the culture had nearly reached to plateau phase. Further increase in the extracellular crude xylanase activity as well as total protein even after cessation of growth could be due to lysis of cells, which led to outflow of proteins into growth medium. Figure S1B in Supplementary Material suggests that maximum growth of WSUCF1 was achieved in ~60 h. Data on crude xylanase activities from WSUCF1 were generated from culture supernatants and under unoptimized medium conditions; therefore, further experiments were performed to optimize the xylanase activity.

### Effects of growth medium pH and temperature on crude xylanase activity

**Figure 1:**
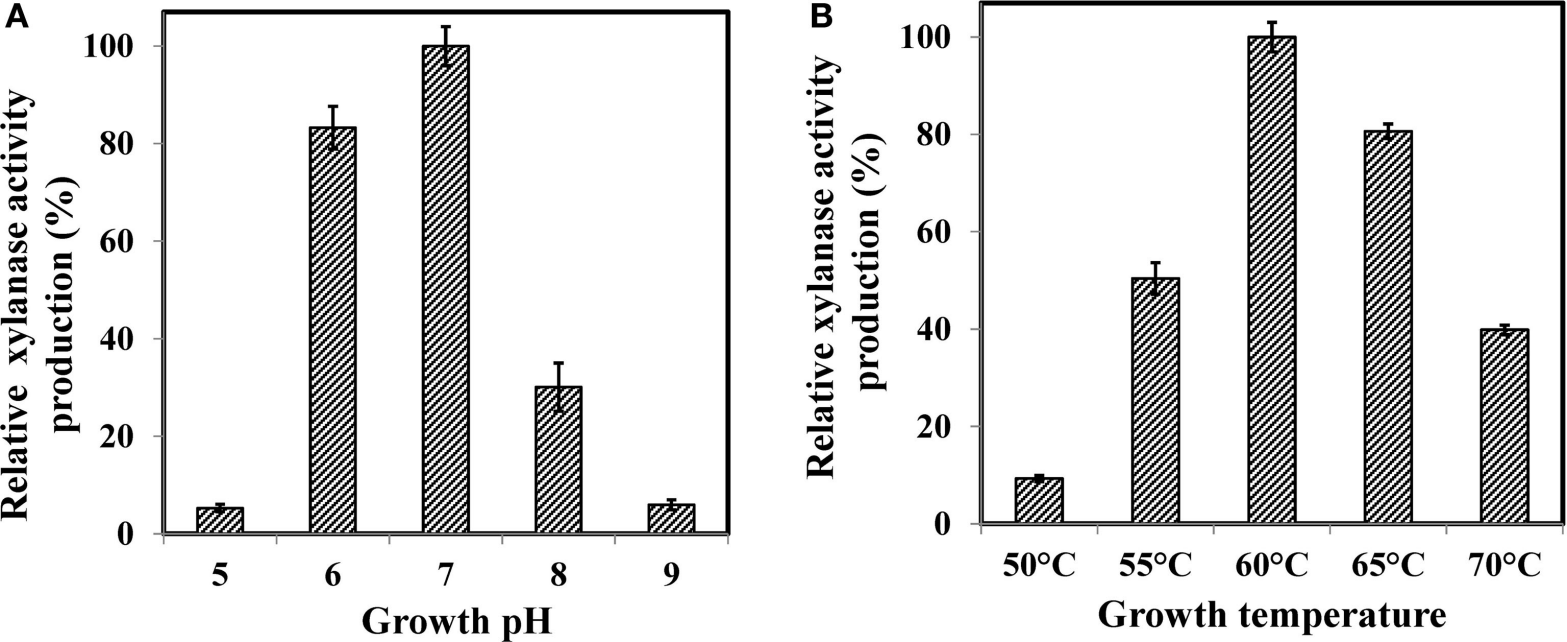
**Effect of (A) growth pH and (B) growth temperature on production of WSUCF1 xylanase**. Enzyme production at optimum pH and optimum temperature was defined as 100% (19.3 and 18.7 U/ml, respectively). Values shown were the mean of duplicate experiments, and the variation about the mean was below 5%.

Effect of growth medium pH is shown in Figure 1A. The pHs 6.0 and 7.0 adequately supported the xylanase activity with maximum at pH 7.0. It declined sharply when the pH was either decreased or increased around the optima. At pH 6.0, relative xylanase activity was 83% and it decreased to 30% at pH 8.0 whereas pH 5.0 and 9.0 did not support the production. It has been shown that growth medium pH strongly influences many enzymatic reactions by affecting the transport of a number of chemical products and enzymes across the cell membrane (Liang et al., 2010). Our results also confirmed that growth medium pH was an important factor affecting the crude xylanase activity in WSUCF1. Optimum xylanase activity near neutrality has been reported earlier for *Bacillus* sp. (Sapre et al., 2005), *Bacillus* SPS-0 (Bataillon et al., 2000), and *Bacillus thermoleovorans* strain K-3d (Sunna et al., 1997).

Growth medium temperature also impacted the production of xylanase activity (Figure 1B). WSUCF1 strain showed maximum amount of xylanase activity at 60°C and the activity decreased drastically when temperature was decreased below 55°C and increased above 65°C. Most of the known xylanase producing microbes have optimum growth pH between 5.5 and 9.5 (Kulkarni et al., 1999). Optimum growth temperature of 60°C has been reported for *Bacillus* SPS-0 (Bataillon et al., 2000) and *Bacillus stearothermophilus* (Khasin et al., 1993). *Bacillus thermantarcticus* (Lama et al., 2004) and *Bacillus* sp. JB 99 (Shrinivas et al., 2010) grew optimally at 65 and 55°C, respectively. Higher growth temperatures are industrially desirable because temperatures above 50°C could lead to significantly reduced risks of mesophilic microbial contamination (Yeoman et al., 2010).

### Effects of different lignocellulosic materials on crude xylanase activity production

**Figure 2:**
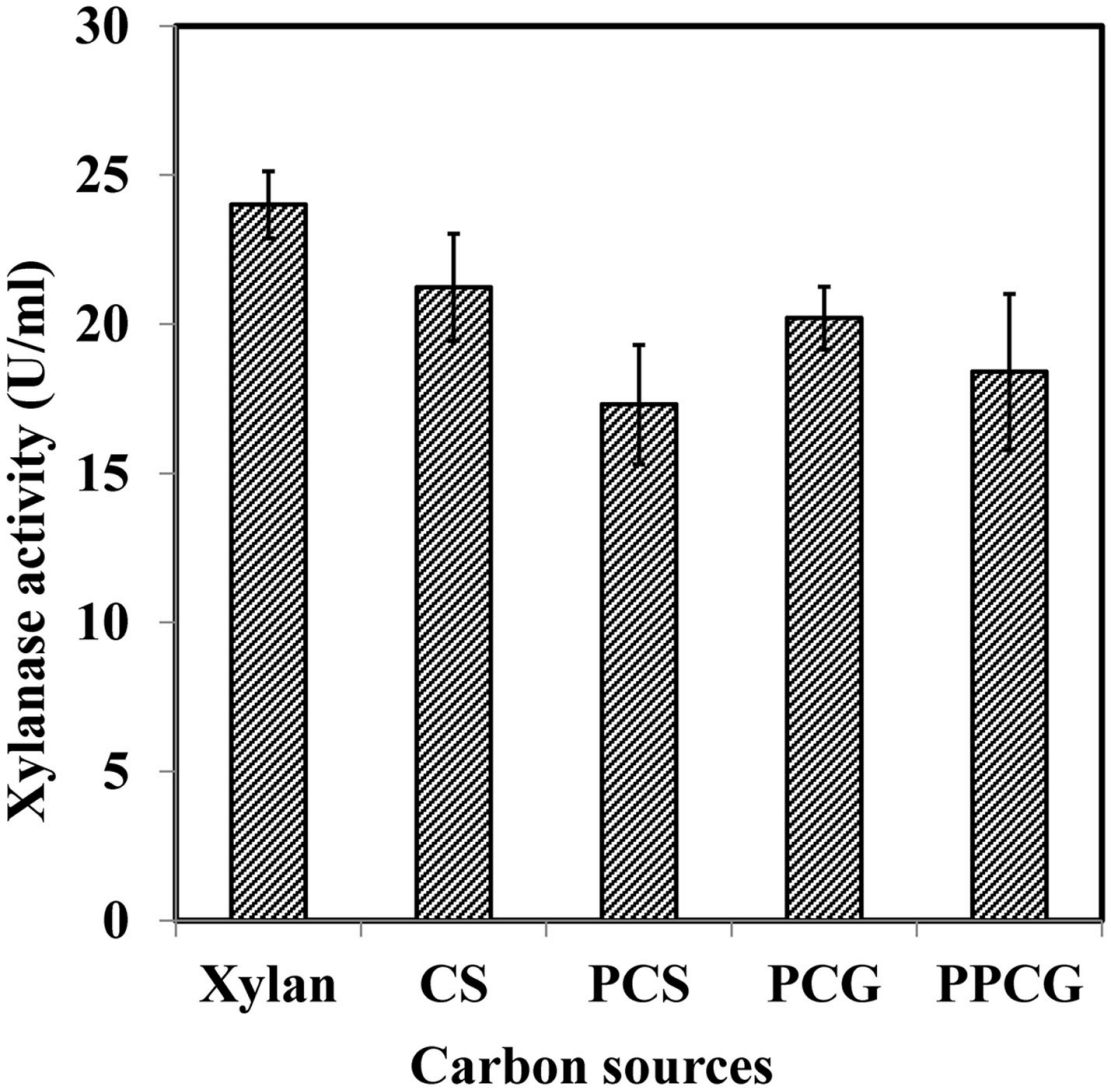
**Effects of xylan and various inexpensive lignocellulosics (prairie cordgrass – PCG, corn stover – CS, pretreated prairie cordgrass – PPCG, and pretreated corn stover – PCS) on xylanase production by WSUCF1**.

The use of inexpensive agriculture residues as substrates for the production of industrial enzymes is a significant way to reduce cost of the overall process. WSUCF1 utilized a variety of inexpensive pretreated as well as untreated cellulosic substrates such as PCG, CS, PPCG, and PCS (Figure 2). Interestingly, in addition to xylan, all lignocellulosic substrates supported for xylanase activity. The crude xylanase activity was maximum (23.8 U/ml, 100%) for Birchwood xylan, followed by untreated CS (88%), untreated prairie cordgrass (84%), PPCG (76%), and PCS (72%). CS contributes roughly up to 80% of all agricultural residues produced in USA therefore considered as a feedstock of choice for various applications, including lignocellulosic ethanol production (Kadam and McMillan, 2003; Zhu et al., 2006; Balan et al., 2009). Kim and Dale (2004) have reported an estimated ethanol production from CS, i.e., 38.4 billion liters of ethanol per year. On the other hand, perennial grasses like prairie cordgrass is also considered as one of the most abundant biomass feedstocks in the Great Plains (Gonzalez-Hernandez et al., 2009; Rastogi et al., 2010; Zambare et al., 2011). Brito-Cunha et al. (2013) reported high xylanases production utilizing sugarcane bagasse and wheat bran as substrates from *Streptomyces* sp. Thermophilic xylanolytic *Thermoanaerobacter* strains have also been reported to produce xylanases with poplar, spruce, miscanthus, wheat straw, whole corn plants, corn cobs, corn stalks, sugarcane bagasse, sweet sorghum, or cotton stalks (Svetlitchnyi et al., 2013).

Literature shows that several microbial species have been reported to use lignocellulosics for xylanase production; however, there are not many reports on utilization of prairie cordgrass and CS as substrates. To reduce the cost of the enzymes needed for the hydrolysis, in-house production of xylanases on various substrates is beneficial. Producing thermostable lignocellulose deconstruction enzymes (crude xylanases) using untreated lignocellulosic biomasses (PCG and PCS) further shows the industrial potential of the WSUCF1 strain. The use of untreated lignocellulosic waste biomasses as substrates for the production of lignocellulolytic enzymes would have both economic and environmental advantages.

### Characterization of thermostable crude xylanase cocktail

**Figure 3:**
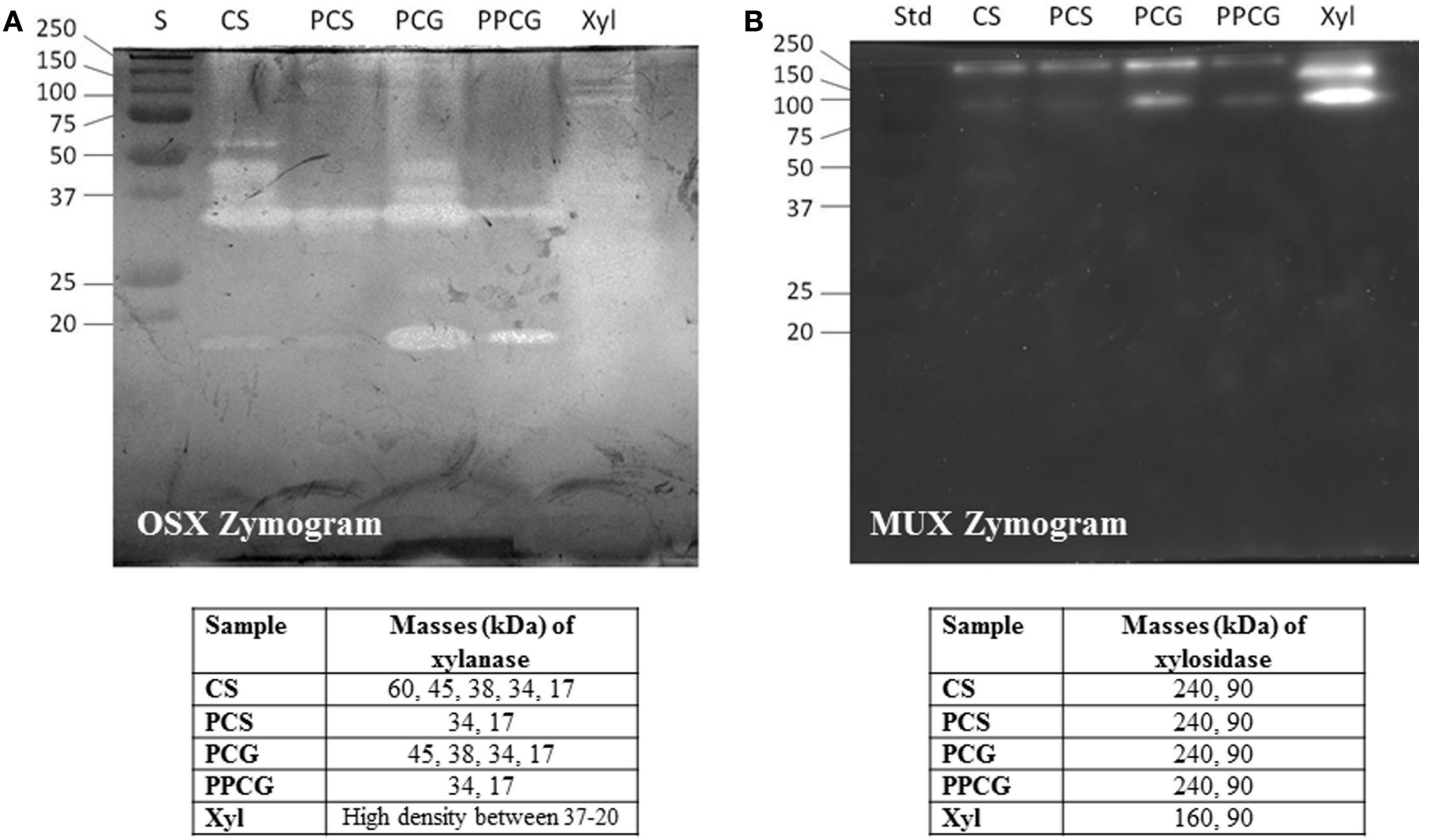
**SDS-PAGE (12%) and zymogram of WSUCF1 (A) endoxylanase activity, (B) β-xylosidase activity**. Lane S, precision plus protein standards (BioRad); Lane CS, corn stover; Lane PCS, pretreated corn stover; Lane PCG, prairie cord grass; Lane PPCG; pretreated prairie cord grass.

Crude xylanases were expressed at high levels in the production medium containing different carbon sources. A SDS zymogram analysis was performed to observe the expression of different xylanases in the crude extract obtained from the growth medium. Figure 3A demonstrates the activity bands for endoxylanase activity on OSX as substrate whereas Figure 3B shows β-xylosidase activity on 4-methylumbelliferyl-β-d-xylopyranoside (MUX) as a specific substrate.

On analyzing the activity bands obtained for endoxylanase activity (Figure 3A), five prominent activity bands were observed at 60, 45, 38, 34, and 17 kDa for the secretome obtained from growth on untreated CS whereas only two activity bands were obtained for PCS secretome, i.e., 34, and 17 kDa. Same trend was noticed for PCG where four activity bands were observed for untreated PCG (45, 38, 34, and 17 kDa) and two activity bands (34 and 17 kDa) for PPCG. On observing the activity bands for Birchwood xylan secretome, a high-density activity band smear was observed between 37 and 20 kDa. The observed trend very well explains the difference in expression of enzymes by a microbe when grown on differently treated complex substrates. On evaluating the activity bands obtained for β-xylosidase activity (Figure 3B), two bands were observed at 240 and 90 kDa for the secretomes obtained from growth on lignocellulosic substrates, whereas interestingly, one different band of 160 kDa along with 90 kDa band was observed for the secretome obtained from growth on pure substrate, i.e., Birchwood xylan. These results demonstrate the importance of variations in the enzyme expression by the same bacterium on different substrates.

Another hypothesis was tested to correlate the enzyme expression observed from zymography experiment with the data obtained on crude xylanase activity (Figure 2). Results showed that higher enzyme units obtained on untreated CS and PCG were positively correlated to the number of active enzymes from untreated CS and PCG zymograms, whereas for pretreated material, lesser enzyme activity was correlated with less enzyme expression.

**Figure 4:**
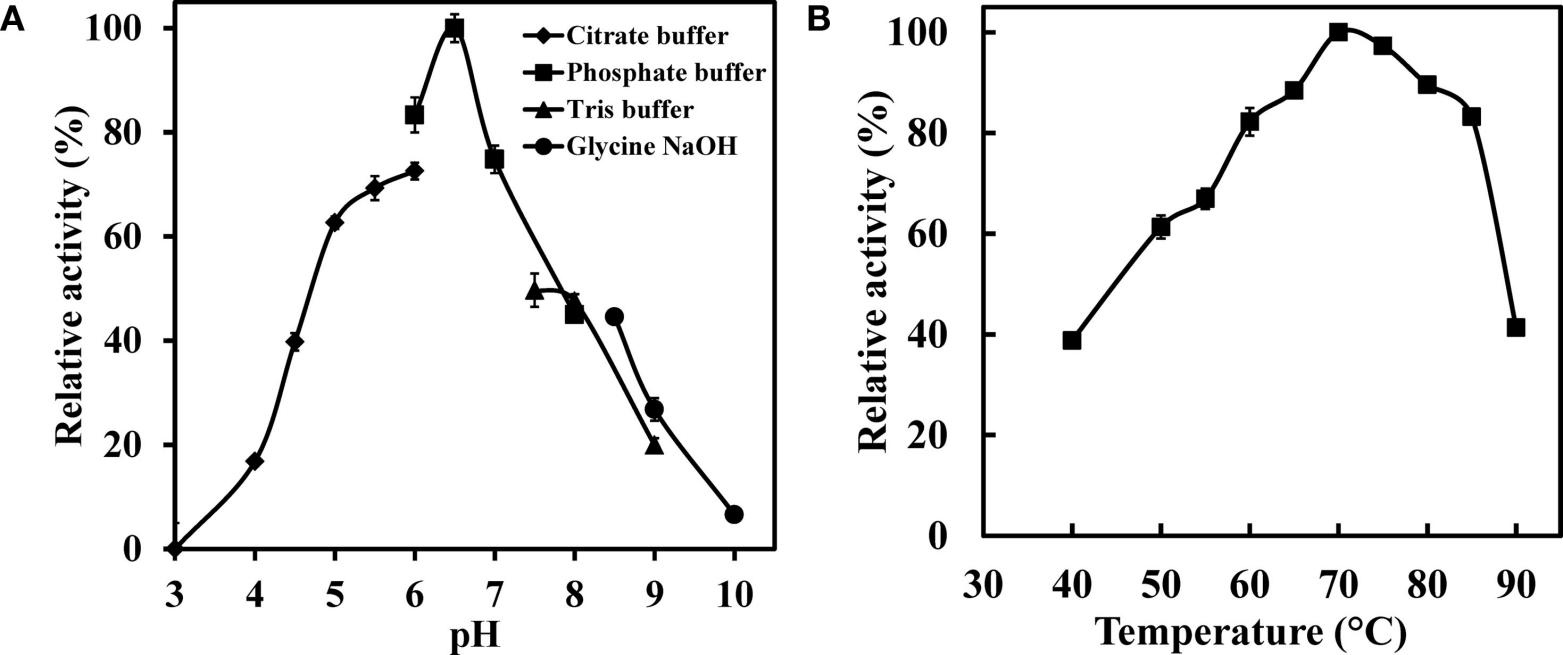
**Impact of (A) pH and (B) temperature on the xylanase activity of WSUCF1 isolate**. The enzyme activity was expressed as percentages of the maximum activity. The points are the averages of triplicates, and error bars indicate ±SDs of the means (*n* = 3). Error bars smaller than the symbols are not shown.

To further characterize the enzyme, effect of the pH on the WSUCF1 crude xylanase activity was examined from pH 3.0 to 10.0 (Figure 4A). WSUCF1 produced maximum xylanase activity in sodium phosphate buffer at pH 6.5. WSUCF1 xylanase exhibited activity in broad pH range of 4.5–8.5 with more than 40% relative activity at pH 4.5 and 8.5. At pH range of 5.5–7.0, more than 70% relative activity was retained. Xylanases from *Thermoanaerobacterium saccharolyticum* NTOU1 (Hung et al., 2011), *Clostridium* sp. TCW1 (Lo et al., 2011), *Actinomadura* sp. S14 (Sriyapai et al., 2011), *Bacillus* sp. (Sapre et al., 2005), *Bacillus flavothermus* strain LB3A (Sunna et al., 1997), and *B. stearothermophilus* T-6 (Khasin et al., 1993) also showed their pH optima at 6.0–7.0.

The impact of different temperatures on the xylanase activity is shown in Figure 4B. Maximum xylanase activity was observed at 70°C. The xylanase activity increased linearly with increasing the temperature up to 70°C and thereafter it declined; however, at 85°C about 83% of its maximum activity was still retained. At lower temperatures, i.e., at 50 and 60°C, 61 and 82% of maximum xylanase activity was observed, respectively. Xylanases from *Geobacillus thermodenitrificans* TSAA1 (Verma et al., 2013), *Bacillus* sp. JB 99 (Shrinivas et al., 2010), *Bacillus licheniformis* 77-2 (Damiano et al., 2006), and *B. flavothermus* strain LB3A (Sunna et al., 1997) also showed their temperature optimum at 70°C. Results demonstrated that WSUCF1 xylanase activity was resistant to change in temperature and hence it is well suited for the harsh process conditions that lignocellulose bioprocessing entails.

**Figure 5:**
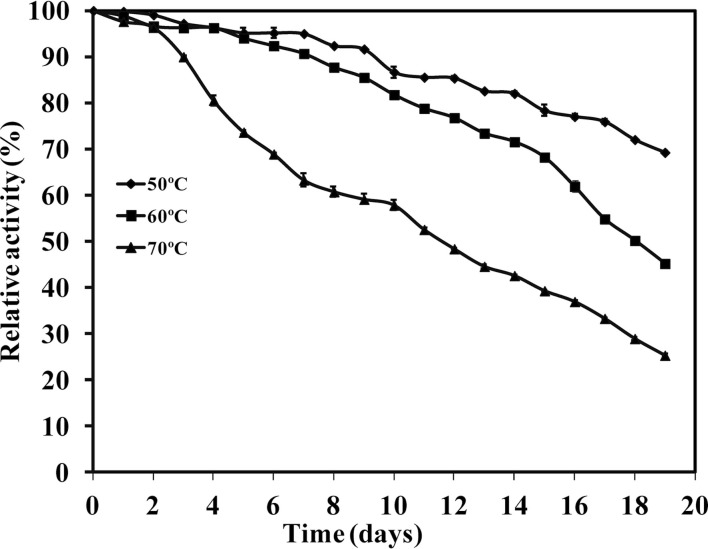
**Thermal stability of xylanase activity produced by WSUCF1 isolate**. The enzyme activities were expressed as percentages of the initial activity. The points are the averages of triplicates, and error bars indicate ±SDs of the means (*n* = 3). Error bars smaller than the symbols are not shown.

Thermal stability profile of WSUCF1 xylanase activity was also observed (Figure 5). Enzyme retained about 70% of its original activity after incubating the enzyme at 50°C for 19 days. At 60 and 70°C, 50% of xylanase activity was retained after incubation for 19 and 12 days, respectively. With further increase in the incubation temperature, decreased enzyme activity was observed. At 80 and 90°C, complete enzyme activity was lost in 150 and 70 min, respectively (data not shown). Xylanase from *G. thermodenitrificans* TSAA1 (Verma et al., 2013) retained >85% activity after exposure to 70°C for only 180 min, xylanase from *Paenibacillus macerans* IIPSP3 exhibited half-life of 6 h at 60°C (Dheeran et al., 2012), *Bacillus subtilis* exhibited half-lives of 16.2, 9.6, and 2.8 h at 60, 70, and 80°C, respectively (Saleem et al., 2012), and *Enterobacter* sp. MTCC 5112 retained 85 and 64% of its activity for 18 h at 60 and 70°C, respectively (Khandeparkar and Bhosle, 2006). These results showed that WSUCF1 xylanase is highly thermostable as compared to the xylanases from other thermophiles. There is considerable interest in enzymes with high thermostability for industrial applications. There had been a number of studies published on enhancing thermal stability using genetic engineering. Stephens et al. (2009) reported the improvement in thermostability of xylanases from *Thermomyces lanuginosus* using error-prone PCR. Jeong et al. (2007) and Zhang et al. (2010) also reported improved thermostability in xylanases using site-directed mutagenesis whereas WSUCF1 is highly thermostable in its native form. Therefore, WSUCF1 xylanase warrants its application in biomass conversion processes, which are carried out at high temperatures.

### Studies on hydrolysis of birchwood xylan

**Figure 6:**
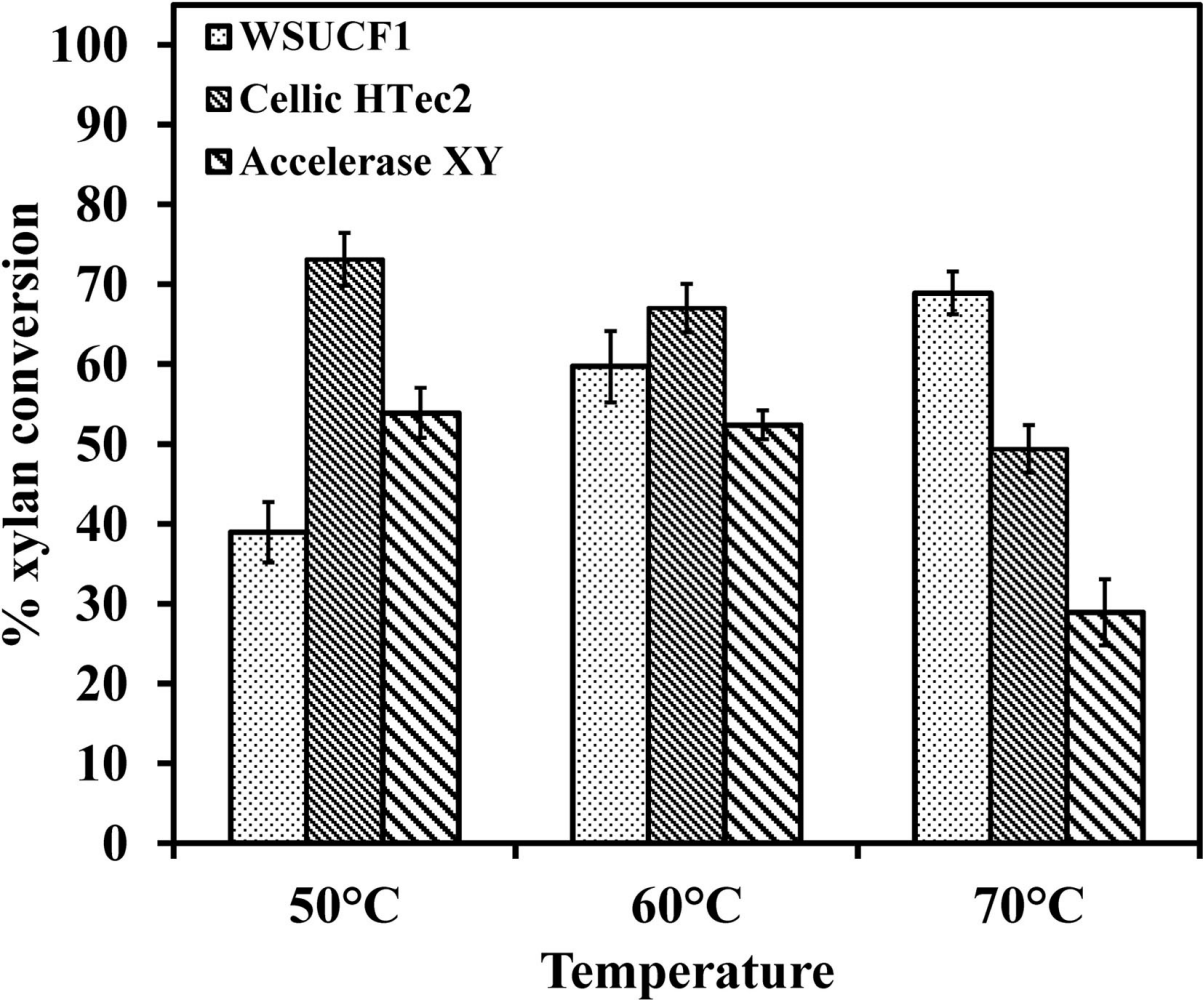
**Hydrolysis of Xylan from Birchwood – comparison of hydrolytic activity of WSUCF1 xylanase, Cellic HTec2, and Genencor Accellerase XY**. The points are the averages of duplicates, and error bars indicate ±SDs of the means (*n* = 2). Error bars smaller than the symbols are not shown.

Thermostable enzymes that hydrolyze lignocellulose to sugars have significant advantages for improving the conversion rate of biomass over their mesophilic counterparts. The hydrolysis rates for WSUCF1 xylanase were higher at 70°C as compared to 50 and 60°C. Figure 6 shows the comparison of hydrolytic capability of WSUCF1 xylanase, Cellic HTec2 (optimum temperature 50°C) and Accellerase XY (optimum temperature 50°C). It can be seen from the figure that in opposed to commercial enzymes, with increase in temperature from 50 to 70°C hydrolysis rates of WSUCF1 xylanase were increased. At 70°C, higher conversions were obtained with WSUCF1 xylanase as compared to Cellic HTEc2 and Accellerase XY after 48 h of incubation. Percentage conversions were calculated on the basis of reducing sugars released from 1 g Birchwood xylan. At 70°C, WSUCF1 xylanase yielded higher conversions of 68.9% as compared to Cellic HTec2 (49.4%) and Accellerase XY (28.92%). However, lower conversion rates were obtained with WSUCF1 as compared to Cellic HTec2 and Accellerase XY at 50°C. The main objective of this experiment was to evaluate the potential of WSUCF1 xylanases at 70°C.

He et al. (2009) reported that the recombinant Xyn2 hydrolyzed <20% of Birchwood xylan when it was incubated at 50°C, but the hydrolysis rates linearly increased with the increasing of reaction time. The xylanase from *B. thermantarcticus* hydrolyzed 70% of Birchwood xylan (2% w/v) within 24 h of reaction time at 70°C, but the hydrolysis rates were <40% at 60 and 80°C (Lama et al., 2004). Our xylan hydrolysis results show that WSUCF1 crude xylanase cocktail performed better than Cellic-HTec2 and Accellerase XY at 70°C. These data suggest that xylanase cocktail produced by WSUCF1 strain is highly active and stable at high temperatures, and therefore, can be used as a part of the enzyme cocktail for efficient hemicellulose hydrolysis.

In summary, thermostable xylanases are the key enzymes for lignocellulosic deconstruction. The existing enzymatic hydrolysis technologies of lignocellulose into sugars, which are carried out at ≤50°C, have several limitations, including very slow enzymatic hydrolysis rates, low yields of sugars from lignocellulose (often incomplete hydrolysis), high dosages of enzymes, and microbial contamination problems. It has repeatedly been suggested that these limitations could be overcome using thermophiles and thermostable enzymes (Viikari et al., 2007; Yeoman et al., 2010; Bhalla et al., 2013, 2014a,b). For instance, Ovissipour et al. (2009) showed that rates of hydrolysis of protein hydrolyzate from Persian Sturgeon (*Acipenser persicus*) using Alcalase^R^ were increased about three-times with increase in temperature from 35 to 55°C. The fact that the strain WSUCF1 thermophile can utilize untreated lignocellulosic substrates to produce xylanases will likely play a key role in reducing production costs of the enzymes. The low-cost enzymes, if made available to biofuel industries, will enable economic conversion of biomass to biofuels. Removal of the CS, PCG, and other biomass wastes will provide a social benefit of protecting the environment and cleaner surroundings. The local CS and prairie grass producers will benefit from an additional and continued income source.

## Conclusions

*Geobacillus* sp. WSUCF1 produced highly active crude xylanase cocktail when grown on inexpensive renewable untreated feedstocks. The xylanase cocktail was found to be highly stable and active over wide range of pH and temperature. Zymography profiles showed an interesting correlation with the enzyme expression and differently treated or untreated lignocellulosic substrates. Higher xylan conversions were obtained at 70°C when compared with commercial enzymes. WSUCF1 crude xylanase is among the most thermostable xylanases produced by thermophilic *Geobacillus* spp. and other thermophilic microbes (optimum growth temperature ≤70°C). These unique properties make WSUCF1 xylanases suitable for thermophilic lignocellulose bioconversion processes.

## Conflict of Interest Statement

The authors declare that the research was conducted in the absence of any commercial or financial relationships that could be construed as a potential conflict of interest.

## Supplementary Material

The Supplementary Material for this article can be found online at http://journal.frontiersin.org/article/10.3389/fbioe.2015.00084
