# Supplementary figures and images for: Highly Thermostable Xylanase Production from A Thermophilic Geobacillus sp. Strain WSUCF1 Utilizing Lignocellulosic Biomass
Source: Front Bioeng Biotechnol. 2015 Jun 16;3:84. doi: 10.3389/fbioe.2015.00084 (PMC4468944; doi:10.3389/fbioe.2015.00084)

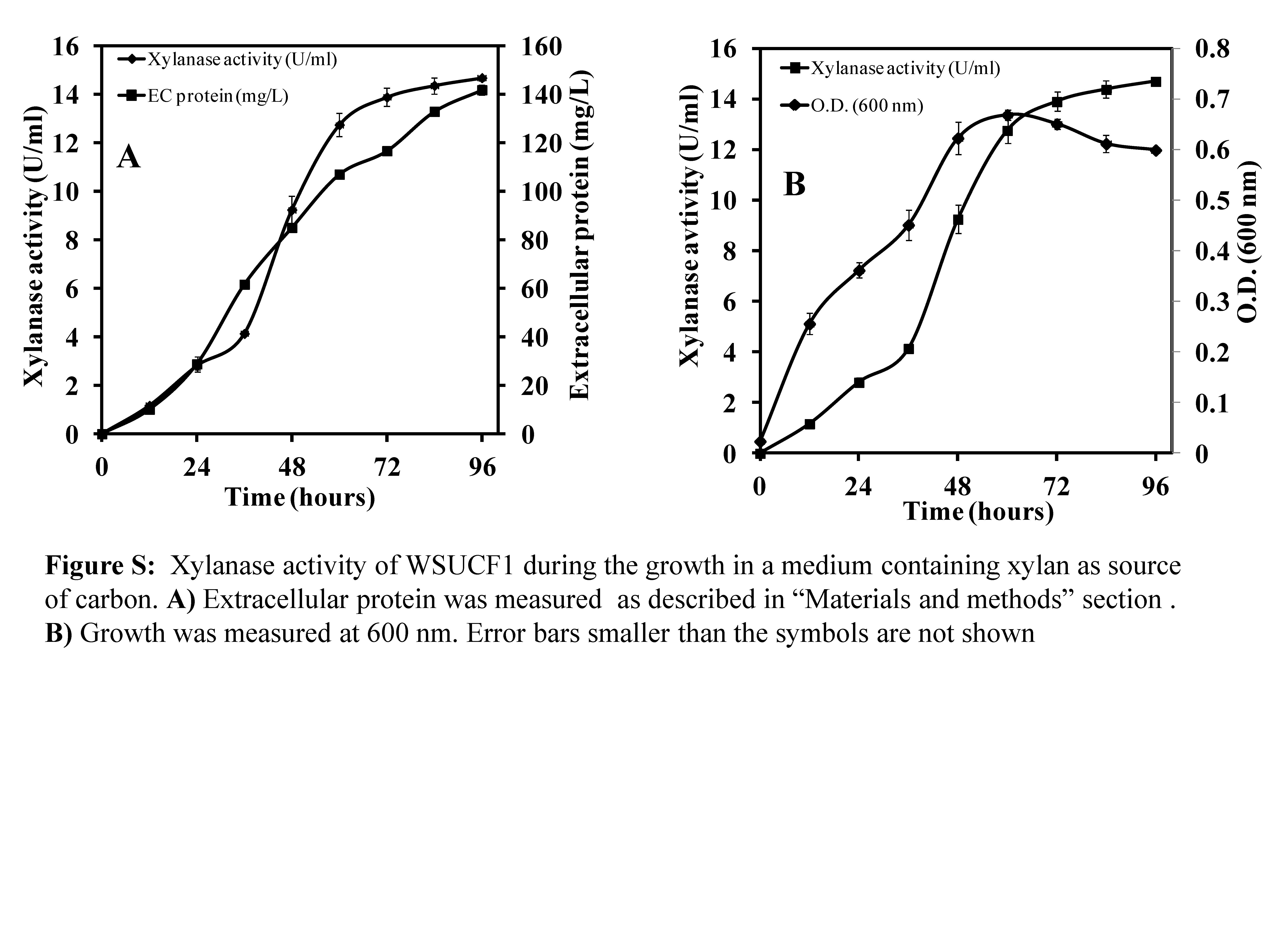

Supplement: Supplementary file 1 [file image_1.tiff]
